# *Magel2* Is Required for Leptin-Mediated Depolarization of POMC Neurons in the Hypothalamic Arcuate Nucleus in Mice

**DOI:** 10.1371/journal.pgen.1003207

**Published:** 2013-01-17

**Authors:** Rebecca E. Mercer, Sheldon D. Michaelson, Melissa J. S. Chee, Tanya A. Atallah, Rachel Wevrick, William F. Colmers

**Affiliations:** 1 Department of Medical Genetics, University of Alberta, Edmonton, Alberta, Canada; 2 Department of Pharmacology, University of Alberta, Edmonton, Alberta, Canada; 3 Beth Israel Deaconess Medical Center, Harvard Medical School, Boston, Massachusetts, United States of America; University of Cambridge, United Kingdom

## Abstract

Prader-Willi Syndrome is the most common syndromic form of human obesity and is caused by the loss of function of several genes, including *MAGEL2*. Mice lacking *Magel2* display increased weight gain with excess adiposity and other defects suggestive of hypothalamic deficiency. We demonstrate *Magel2-*null mice are insensitive to the anorexic effect of peripherally administered leptin. Although their excessive adiposity and hyperleptinemia likely contribute to this physiological leptin resistance, we hypothesized that Magel2 may also have an essential role in intracellular leptin responses in hypothalamic neurons. We therefore measured neuronal activation by immunohistochemistry on brain sections from leptin-injected mice and found a reduced number of arcuate nucleus neurons activated after leptin injection in the *Magel2*-null animals, suggesting that most but not all leptin receptor–expressing neurons retain leptin sensitivity despite hyperleptinemia. Electrophysiological measurements of arcuate nucleus neurons expressing the leptin receptor demonstrated that although neurons exhibiting hyperpolarizing responses to leptin are present in normal numbers, there were no neurons exhibiting depolarizing responses to leptin in the mutant mice. Additional studies demonstrate that arcuate nucleus pro-opiomelanocortin (POMC) expressing neurons are unresponsive to leptin. Interestingly, *Magel2*-null mice are hypersensitive to the anorexigenic effects of the melanocortin receptor agonist MT-II. In Prader-Willi Syndrome, loss of *MAGEL2* may likewise abolish leptin responses in POMC hypothalamic neurons. This neural defect, together with increased fat mass, blunted circadian rhythm, and growth hormone response pathway defects that are also linked to loss of *MAGEL2*, could contribute to the hyperphagia and obesity that are hallmarks of this disorder.

## Author Summary

Prader-Willi Syndrome (PWS) is a genetic condition that causes insatiable appetite and severe obesity in affected children. Several genes are inactivated in children with PWS, but no one knows which gene is important for normal body weight. One of the inactivated genes is called *MAGEL2*. We previously found that mice missing the equivalent mouse gene, named *Magel2*, have more fat and are overweight compared to mice with an intact *Magel2* gene. In other forms of genetic childhood obesity, there are deficiencies in the way that the brain senses a hormone called leptin, which is made by fat cells. In this study, we show that mice lacking *Magel2* are defective in their ability to sense leptin. We identified the specific type of brain cell that should become activated when treated with leptin, but that is not activated in mice lacking *Magel2*. We then found that we could bypass this leptin insensitivity by administering a drug that compensates for the lack of activity of these neurons. We propose that loss of the *MAGEL2* gene in people with Prader-Willi Syndrome may cause deficient leptin sensing, leading to the increased appetite and obesity that are hallmarks of this genetic condition.

## Introduction

Energy balance is regulated in part by the coordinated action of specialized neurons within the hypothalamus of the brain, which sense circulating signals of energy stores such as the adipocyte derived hormone, leptin [1]. The arcuate nucleus (ARC) is a key hypothalamic region involved in energy balance regulation, and is a major site for leptin action. Two distinct populations of ARC neurons, expressing either Neuropeptide Y (NPY) and Agouti-related peptide (AgRP) or pro-opiomelanocortin (POMC), have opposing effects on energy balance. NPY and AgRP, *via* different mechanisms, stimulate food intake and reduce energy expenditure, with the overexpression of either leading to obesity [2]–[4]. In contrast, POMC is processed into several shorter peptides including α-MSH, which reduces food intake and stimulates energy expenditure through melanocortin-responsive neurons in the paraventricular nucleus and elsewhere [5]. Mutations that affect processing or lead to loss of expression of the POMC gene also cause obesity in mice and humans [6]–[8].

Impaired hypothalamic regulation of energy balance is found in numerous genetic forms of human obesity, including congenital deficiency of leptin (MIM 164160) [9], leptin receptor mutations (MIM 601007) [10], MC4R melanocortin receptor mutations (MIM 601665) [11], and Bardet-Biedl Syndrome (MIM 209900) [12]. Impaired energy homeostasis may also contribute to the severe hyperphagia and obesity seen in people with Prader-Willi Syndrome (PWS, MIM 176270), the most common genetic form of syndromic obesity in humans [13]. People with PWS typically have a loss of function of several contiguous genes, including *MAGEL2*, a member of the melanoma antigen (MAGE) family of proteins [14]. MAGE proteins act in intracellular signaling pathways that modulate protein modification, protein degradation, cytoskeletal rearrangement, and transcription [15]. In mice, *Magel2* is predominantly expressed in the central nervous system, with highest expression levels in the hypothalamus [16], [17]. We previously showed that gene-targeted mice lacking *Magel2* become overweight with increased adiposity as adults [18], and exhibit delayed puberty, irregular estrous cycles, and early onset infertility [19]. As obesity and infertility are common in animal models with impaired leptin responses [20], we hypothesized that *Magel2*-null mice may also respond abnormally to leptin. We now report that *Magel2-*null mice display physiological leptin resistance, that leptin resistance precedes the development of increased adiposity, and that leptin-mediated electrophysiological responses in POMC neurons are conspicuously absent in these animals.

## Results

### *Magel2-*Null Mice Lose Less Weight during a Fast, and Eat Less Food and Gain Less Weight after Fasting

**Figure 1:**
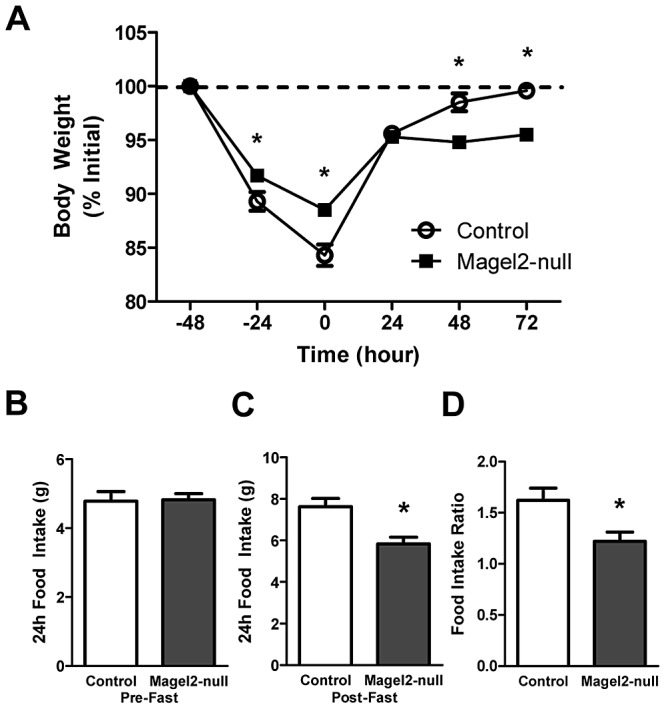
*Magel2*-null mice have abnormal weight recovery and compensatory refeeding after fasting. A) Body weights of adult male mice subjected to a 48 h fast and a 72 h refeeding period. *Magel2*-null mice lost less weight while fasting, and recovered less weight during refeeding (*P<0.05, compared between genotypes by Student's *t*-test). B) 24 h food intake pre-fast and C) post-fast (*P<0.01). D) *Magel2*-null mice have a reduced food intake ratio - the ratio of food consumed after fasting to food consumed before fasting - compared to controls (*P<0.05). *n* = 6 mice of each genotype. Values are means ± SEM.

Leptin maintains homeostatic control of weight, regulating ingestive behavior and energy expenditure in response to changes in nutritional energy availability. The fall in circulating leptin that occurs with food deprivation normally causes increased feeding when food is reinstated, restoring normal weight and fat mass [1]. However, refeeding-associated weight gain and hyperphagia are dysregulated in mice with diet-induced obesity [21] or mice carrying mutations that selectively ablate POMC neurons [22], [23] or that decrease levels of hypothalamic neuropeptides [24], [25]. To determine if Magel2 is important for compensatory responses after fasting, we subjected mice to a prolonged (48 h) fast. While control mice lost 16% of their body weight after fasting, *Magel2*-null mice lost significantly less body weight (12% of initial weight), consistent with their previously noted reduced locomotor activity (Figure 1A). We then refed the fasted mice, and measured food intake and body weight over the next 3 days. Body weight returned to baseline within 2 days of refeeding in control mice, but *Magel2*-null mice remained underweight even after 3 days (Figure 1A). Food intake was similar before fasting (Figure 1B), but *Magel2-*null mice ate less food during the initial 24 h recovery period (Figure 1C), resulting in a significantly reduced food intake ratio - the ratio of food consumed after fasting to food consumed before fasting - compared to control mice (Figure 1D). These results suggest that the hypothalamic pathways required for compensatory refeeding are defective in *Magel2-*null mice.

### *Magel2*-Null Mice Lack the Anorexigenic Response to Peripherally Administered Leptin

**Figure 2:**
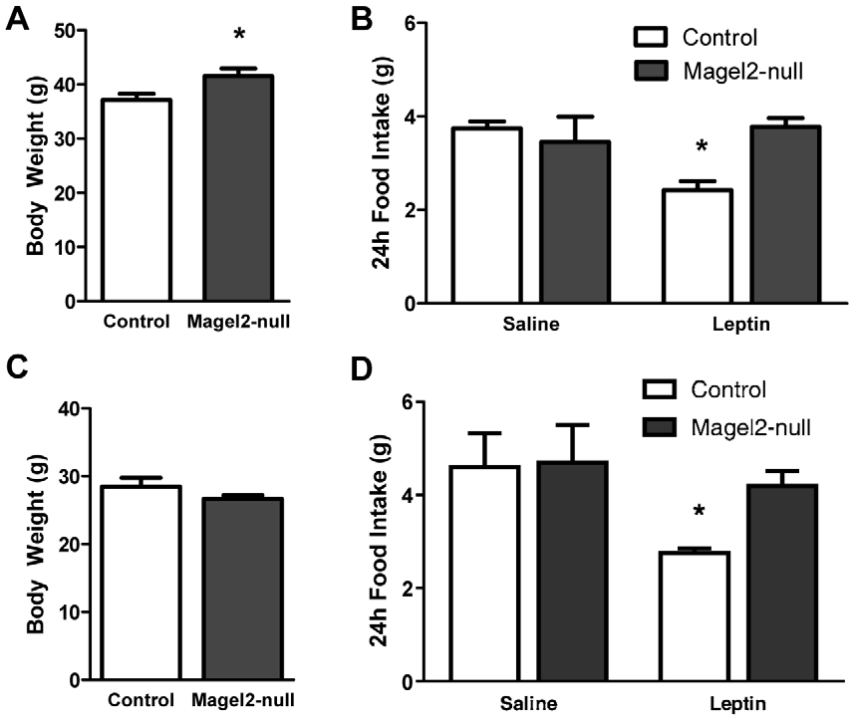
Peripherally administered leptin fails to reduce food intake in *Magel2*-null mice. A) Body weights of 20 week old male mice (*P<0.05, compared between genotypes by Student's *t*-test), and B) 24 h food intake following leptin or PBS (saline) injections. Leptin reduced food intake in control mice (*P<0.005, compared before and after treatment), while leptin-treated *Magel2*-null mice showed no significant change in food intake. *n* = 10 of each genotype. C) Body weights of 6 week old male mice and D) 24 h food intake following leptin or PBS injections. Leptin reduced food intake in control (*P<0.05, compared before and after treatment) but not *Magel2*-null mice. Controls, *n* = 7, *Magel2-*null, *n* = 6. Values are means ± SEM.

*Magel2-*null mice have excess adipose tissue, and high levels of circulating leptin suggesting reduced leptin sensitivity [18]. At 20 weeks of age, *Magel2*-null mice are 14% heavier than control mice (Figure 2A). To examine whether *Magel2*-null mice are sensitive to exogenous leptin, we measured food intake in singly housed male mice using a crossover study design in which the same animals received either intraperitoneal (ip) leptin (2.5 mg/kg) or phosphate buffered saline (PBS) approximately 1 week apart. In control leptin-treated mice, food intake was reduced by about 30% in the 24 h following leptin injection, as expected. However, leptin-treated *Magel2*-null mice showed no reduction in food intake following ip leptin (Figure 2B). Decreased sensitivity to peripherally administered leptin can occur in mice with diet-induced obesity that have very high (e.g. ten-fold elevated) leptin levels even in the absence of a genetic mutation [26], [27]. In contrast, *Magel2* mice typically have only two-fold elevated leptin even as older adults. Nonetheless, we tested leptin sensitivity in younger (6-week old) mice, where there is no difference in body weight between *Magel2*-null and control animals (Figure 2C). Leptin treatment in young control mice again caused a reduction of approximately 35% in 24 h food intake compared to PBS treatment. In contrast, there was no reduction in 24 h food intake in leptin-treated young *Magel2*-null mice (Figure 2D). These results suggest that *Magel2*-null mice that are similar in weight to controls are nevertheless insensitive to the anorexigenic effect of peripherally administered leptin.

### *Magel2* Deficiency Reduces Leptin-Mediated Phosphorylation of STAT3 and Induction of c-Fos Expression in the Arcuate Nucleus

**Figure 3:**
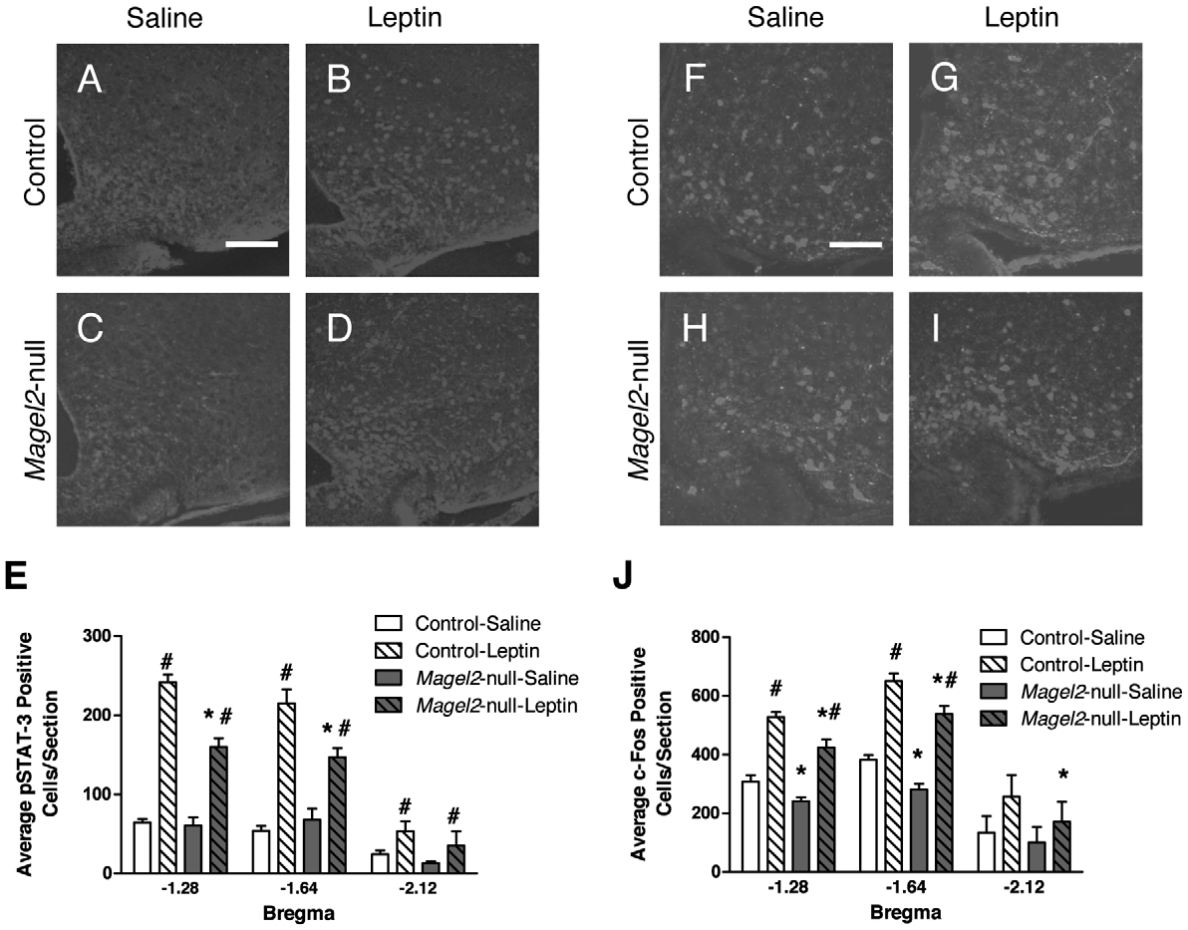
pSTAT3 and c-fos expression in ARC neurons in leptin-treated mice. A–D) Representative immunohistochemistry (IHC) images showing pSTAT3 immunoreactivity following ip PBS (saline) or leptin injection. Scale bar, 100 µm. E) *Magel2*-null mice have fewer pSTAT3 positive cells than control following leptin injection (Bregma −1.28, *P*<0.001; Bregma −1.64, *P*<0.005; Bregma −2.12, *P*>0.5, *compared between genotypes by Student's *t*-test) but both genotypes have more pSTAT3 positive cells after leptin treatment than after PBS (#, *P*<0.05, compared between treatments by Student's *t*-test; *n* = 8 mice of each genotype). F–I) Representative images of c-fos IHC following ip PBS and leptin injection. Scale bar 100 µm. J) Leptin induced c-fos expression in both *Magel2*-null and control mice compared to PBS (#*P*<0.05, compared between treatments by Student's *t*-test). At both baseline and following leptin treatment, *Magel2*-null mice had significantly fewer c-fos positive cells compared to controls (Bregma −1.28 ∼18% reduction, Bregma −1.64 ∼20% reduction, Bregma −2.12 ∼35% reduction, *P<0.05, compared between genotypes by Student's *t*-test). Values in E) and J) are means ± SEM.

We next examined the activation of the leptin receptor by measuring levels of phosphorylated Signal Transducer and Activator of Transcription 3 (pSTAT3) [28], [29] in the ARC following a single ip leptin (2.5 mg/kg) injection. While few pSTAT3-positive neurons were seen in the ARC following PBS injection in both *Magel2*-null and control animals (Figure 3A, 3C, 3E), numerous pSTAT3-positive cells were seen in the ARC of both genotypes after leptin injection (Figure 3B, 3D). Nonetheless, detailed cell counts throughout the ARC revealed a 30–35% reduction in pSTAT3-positive cells in leptin-injected *Magel2*-null mice compared to leptin-injected control (Figure 3E). Next, we measured the induction of c-fos, an immediate early gene marker of neuronal activation that is detected in POMC but not NPY neurons in the ARC after leptin injection [30], [31]. Baseline c-fos immunoreactivity was observed in PBS-injected control animals (Figure 3F, 3H, 3J), and leptin treatment caused a significant increase in c-fos expression in both control and *Magel2*-null mice (Figure 3G, 3I, 3J), particularly in more posterior regions of the ARC where the majority of leptin-sensitive POMC neurons are located [32]. Interestingly, fewer c-fos positive cells were observed in *Magel2-*null mice after either PBS or leptin injection compared to similarly treated control mice (Figure 3J).

### *Magel2*-Null Mice Have Fewer ARC POMC Neurons

**Figure 4:**
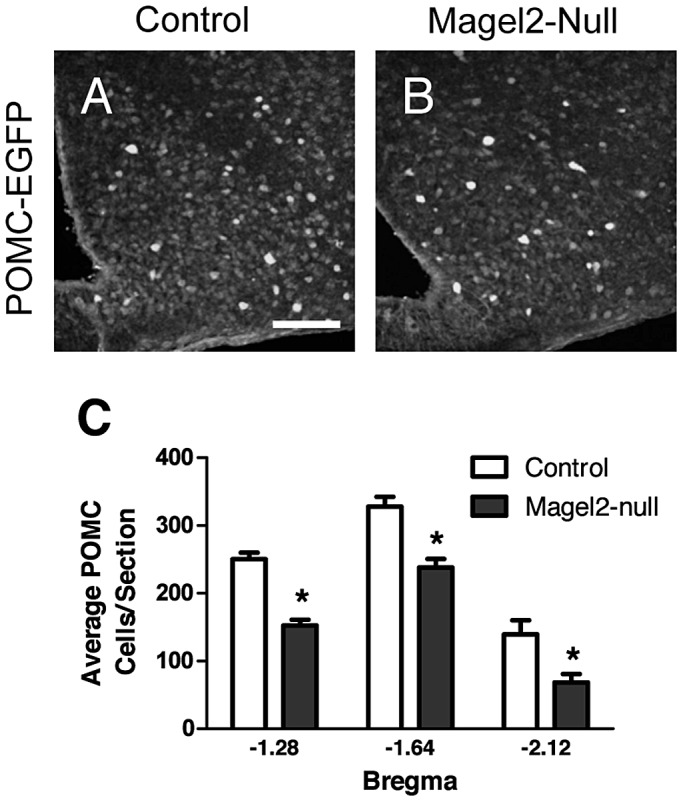
*Magel2*-null mice have fewer ARC POMC neurons. A–B) Representative images of GFP (POMC) IHC in A) control and B) *Magel2*×POMC^EGFP^ mice. Scale bar 100 µm. C) *Magel2*×POMC^EGFP^ mice have fewer GFP expressing (POMC) cells at all levels of the ARC (Bregma −1.28, 39% reduction, *P<10^−8^; Bregma −1.64, 27% reduction, *P<10^−5^; Bregma −2.12, 52% reduction, *P<0.01), compared between genotypes by Student's *t*-test. Values are means ± SEM.

POMC neurons form an important part of the hypothalamic energy balance neural circuitry, and are activated in response to leptin [33]. Fewer leptin-induced pSTAT3 and c-fos immunoreactive cells were observed in the ARC of *Magel2*-null mice, particularly in areas previously shown to contain higher levels of leptin-sensitive POMC neurons. We therefore counted POMC/EGFP-positive neurons in the ARC of *Magel2*×POMC^EGFP^ and control mice, and found on average 39% fewer POMC+ neurons in the *Magel2*-null mice than in controls (Figure 4). This reduction was most evident in the more posterior region of the ARC, where 52% fewer POMC+ cells were found (Figure 4C). The number of LepRb positive neurons (measured as EGFP positive cells in the ARC of offspring from a *Magel2*×LepRb^EGFP^ cross) did not differ between mutants and controls. Thus, loss of POMC neurons can partially explain the reduction seen in leptin-induced pSTAT3 and c-fos expression in the ARC of *Magel2-*null mice. Alternatively, it is possible that these neurons are still present, but that the expression of POMC/EGFP has fallen below the detection limit of this experiment.

### Leptin Fails to Activate POMC Neurons in the ARC of *Magel2-*Null Mice

**Figure 5:**
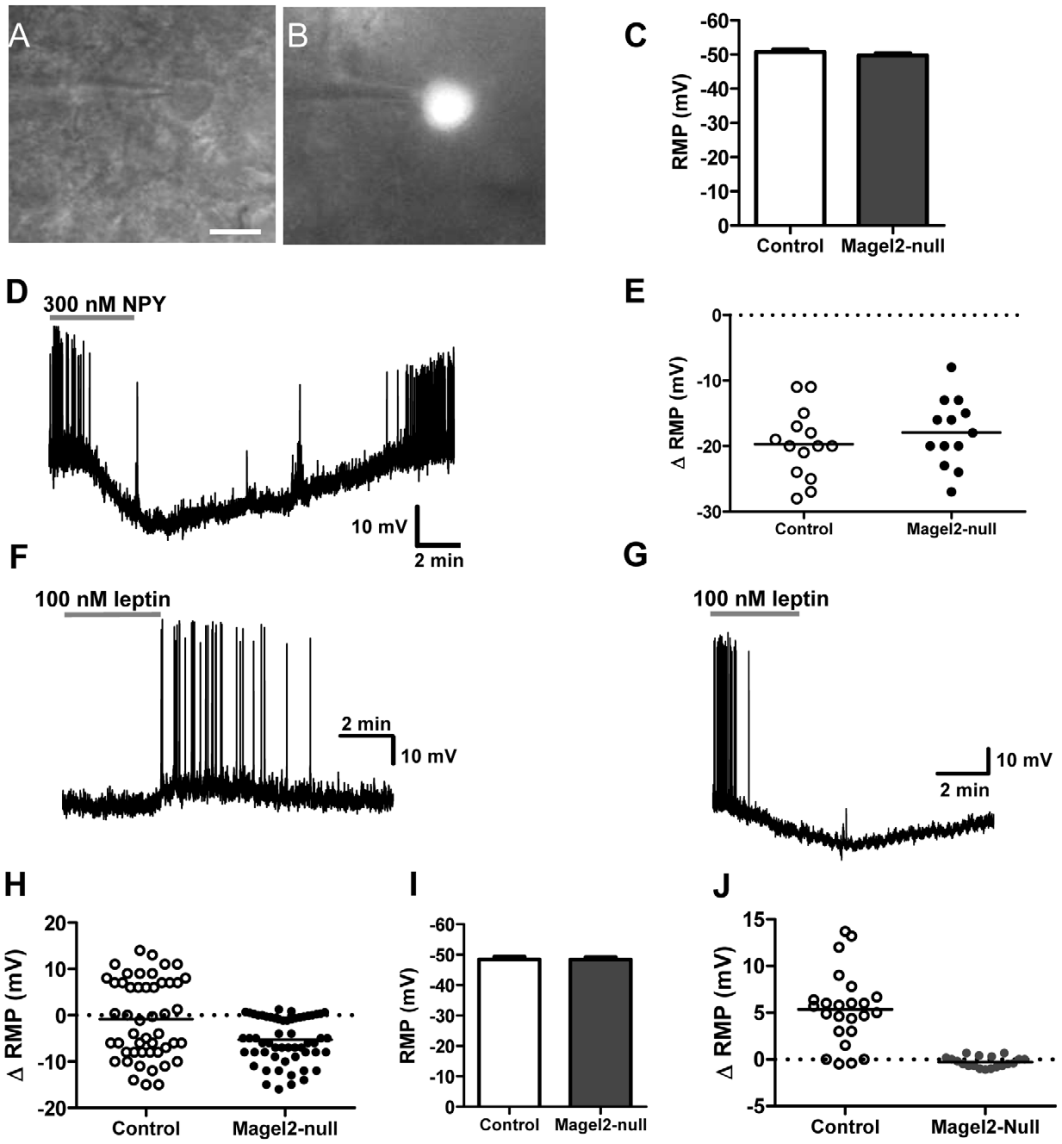
Magel2 is required for the leptin-induced depolarizing response in POMC neurons. Example of a LepRb^EGFP^ positive neuron identified for electrophysiological recordings using: A) infrared-differential interference contrast imaging (scale bar, 10 µm) and B) epifluorescence. C) Mean RMP of ARC LepRb+ neurons (*n*>50 of each genotype, values are means ± SEM). D) Current clamp recording of a LepRb^EGFP^ neuron showing the hyperpolarizing effect of 300 nM NPY. E) There was no difference in the magnitude of hyperpolarization between control and *Magel2*-null neurons treated with 300 nM NPY. F–H) Current clamp recordings of typical responses to 100 nM leptin in F) depolarizing neurons (ΔRMP>2 mV over baseline), and G) hyperpolarizing neurons (ΔRMP>2 mV below baseline). H) Changes in RMP with application of 100 nM leptin to ARC LepRb^EGFP^ neurons. Circles represent individually tested neurons. Depolarizing, hyperpolarizing, and unresponsive neurons were found in control slices, while only hyperpolarizing and unresponsive neurons were found in *Magel2*-null slices. Difference between genotypes is significant by Fishers Exact Test, *P*<10^−8^. I) Mean RMP of ARC POMC^EGFP^ neurons (*n*>40 of each genotype, means ± SEM). J) Changes in RMP caused by application of 100 nM leptin to ARC POMC^EGFP^ neurons. Depolarizing responses were observed in control but not *Magel2*-null neurons. Difference between genotypes in the number of depolarizing neurons is significant by Fishers Exact Test, *P*<10^−8^.

To directly examine leptin responses in ARC neurons, we performed whole-cell visualized-patch recordings of fluorescent neurons in mice expressing enhanced GFP in leptin receptor-positive (LepRb+) neurons (Figure 5A, 5B). First, the resting membrane potential (RMP) of LepRb+ neurons (Figure 5C) and the input resistance (data not shown) were comparable between *Magel2*×LepRb^EGFP^ and control mice. NPY hyperpolarizes the majority of leptin-responsive ARC cells (Figure 5D) [34]. Application of either 100 nM (data not shown) or 300 nM NPY produced a robust hyperpolarization of virtually all ARC LepRb+ neurons tested in both *Magel2*-null and control slices, indicating that Magel2 is not required for normal NPY signaling (Figure 5E). We then examined the leptin (100 nM) responses in LepRb+ neurons in the ARC. Leptin normally activates (depolarizes) POMC neurons, and inhibits (hyperpolarizes) NPY neurons [33], [35], so we expected to observe both responses in the mixed neuronal populations represented by LepRb+ cells in the ARC. Leptin induced both hyperpolarizing and depolarizing responses in LepRb+ cells in slices from control mice, with a few unresponsive cells (Figure 5F–5H). All cells tested, including leptin-unresponsive cells, exhibited a normal electrophysiological response to 300 nM NPY. In striking contrast, LepRb+ cells in slices from *Magel2*-null mice never exhibited depolarizing responses to leptin. In these slices, leptin-mediated hyperpolarizing responses were seen at a frequency comparable to controls, while more leptin-unresponsive cells (which nevertheless showed normal NPY responses) were found (Figure 5H). These results suggest that the inhibitory action of leptin is unaffected at ARC LepRb+ neurons of *Magel2-*null mice, but that the excitatory effect of leptin, typically observed at POMC neurons, is selectively absent.

To more directly examine the population of neurons specifically activated by leptin in the ARC, we identified POMC neurons using crosses with mice expressing GFP in POMC cells (*Magel2*×POMC^EGFP^ and littermate controls). As with LepRb+ neurons in the ARC, POMC+ neurons from control and *Magel2*-null animals did not differ in their RMP (Figure 5I). We then tested leptin (100 nM) responses in POMC+ cells located in the posterior and medial regions of the ARC, where a large number of POMC neurons are leptin-sensitive [32]. Leptin induced a depolarization in the majority of POMC neurons from control mice, but no depolarizing effects were seen in response to leptin in POMC neurons of *Magel2-*null mice. This confirms that POMC neurons in these animals are insensitive to the acute administration of leptin (Figure 5J).

**Figure 6:**
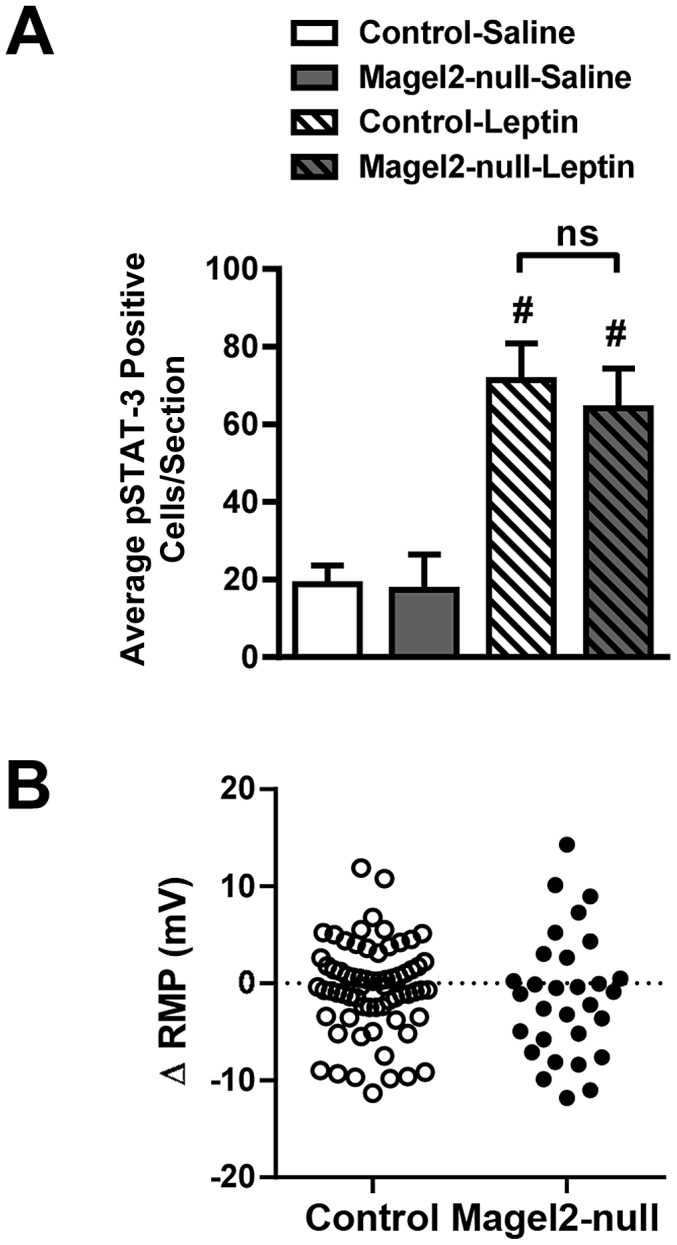
Magel2 is not required for leptin-mediated responses in the VMN. A) While injection of leptin induced a significant increase in pSTAT3 immunoreactivity compared with saline throughout the entire VMN both in control and *Magel2*-null mice, there was no difference between genotypes in cell numbers demonstrating pSTAT3 immunoreactivity in response to leptin injection. Values are means ± SEM, ^#^ *P*<0.05, compared within genotypes for saline vs. leptin treatment (Student's *t*-test). B) Changes in RMP with application of 100 nM leptin to VMN LepRb^EGFP^ neurons. Data points represent individually tested neurons (total *n* = 68 in control, *n* = 30 in *Magel2*-null). Depolarizing, hyperpolarizing, and unresponsive neurons were found in both control and *Magel2*-null slices. There was no significant difference between genotypes in the populations of depolarizing or hyperpolarizing responses to leptin application (Fishers Exact Test, *P*>0.2).

In addition to the ARC POMC neurons, many other neurons in the hypothalamus are depolarized by leptin [36], [37]. To determine the specificity of the effect of *Magel2* loss on depolarizing actions mediated by the leptin receptor, we studied leptin responses in the ventromedial hypothalamic nucleus, which comprise both depolarizing and hyperpolarizing responses [38]. The serial microscope sections stained for pSTAT3 used in the experiments on ARC above were re-imaged for the VMN using confocal microscopy, and pSTAT3-positive neurons were counted. Leptin treatment caused an increase in numbers of neurons immunopositive for pSTAT3, but in contrast to results for ARC, no significant differences in numbers of pSTAT3 neurons were seen in the *Magel2-*null animals compared to controls (Figure 6A). Electrophysiological recordings were made from neurons in the dorsomedial and central VMN as described [39] in slices prepared from *Magel2*-null×LepRb^EGFP^ and LepRb^EGFP^ control animals. In VMN from control LepRb^EGFP^ mice, we observed a mixture of depolarizing and hyperpolarizing responses to 100 nM leptin, along with unresponsive neurons. In contrast to the results in ARC, there were no significant differences in the numbers of neurons depolarized or hyperpolarized by leptin (Figure 6B). Thus, leptin-mediated depolarization of VMN neurons is unaffected by the loss of *Magel2*.

### *Magel2*-Null Mice Are Hypersensitive to the Melanocortin Agonist MT-II

**Figure 7:**
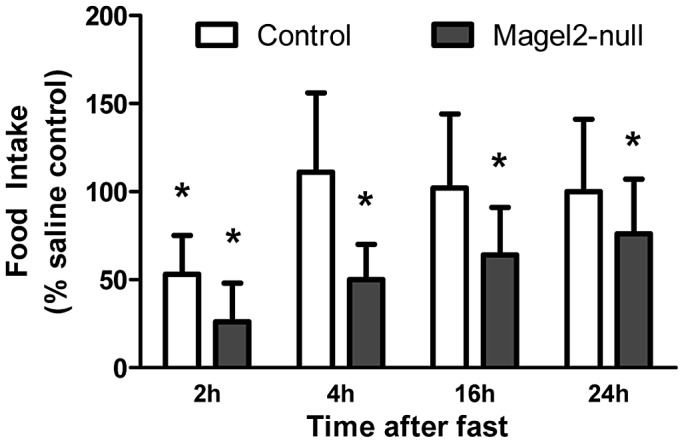
Effects of intraperitoneal MT-II on food intake. Food intake following 2.5 mg/kg injection of MT-II or saline in 24 h fasted mice. MT-II significantly reduced food intake for the first 2 h of refeeding in control mice, but this effect was no longer present by 4 h. In *Magel2*-null mice, MT-II reduced food intake to a greater extent and this effect was still evident 24 h after injection. *n* = 6 mice of each genotype. Values are means ± SEM. *P<0.05, compared to saline-injected (100%) food intake within each genotype by Student's *t*-test.

A failure of POMC neurons to depolarize in response to leptin application is predicted to cause loss of α-MSH release. In other animal models of leptin insensitivity, an enhanced response to the direct application of either α-MSH or the synthetic melanocortin agonist MT-II is observed [40]–[42]. We therefore examined the effect of MT-II on food intake in *Magel2*-null mice. Mice were fasted for 24 h, and then injected with MT-II (2.5 mg/kg ip). Compared to PBS-injected control fasted mice, MT-II-injected control fasted mice consumed 50% less food over the first 2 h of refeeding. After this time, there was no significant difference in food intake between control mice injected with MT-II or PBS. In contrast, *Magel2-*null mice injected with MT-II had a greater reduction in food intake compared to PBS injection, and this decrease was still evident after 24 h (Figure 7). This result suggests that the melanocortin system is chronically upregulated in *Magel2*-null mice, likely as a result of the loss of melanocortinergic tone from ARC POMC neurons.

## Discussion

Mice lacking *Magel2* have increased adiposity with proportionately increased leptin, suggesting leptin insensitivity [18], [43]. Here, we show that *Magel2*-null mice are physiologically resistant to the effects of exogenously applied leptin, both before and after the onset of increased adiposity. Further, this leptin resistance is accompanied by a 39% reduction in the number of POMC neurons in the ARC, and by a complete absence of leptin-induced depolarization responses in the remaining POMC neurons. Magel2 is therefore essential for normal leptin signaling in POMC neurons, and for the differentiation, proliferation, or survival of this population of neurons. Interestingly, the effect of *Magel2* loss on leptin-mediated depolarization is not universal, even within the hypothalamus, as equivalent numbers of energy balanced-related VMN neurons not only exhibit pSTAT3 immunoreactivity, but also equal numbers are depolarized in the Magel2-null animals. Loss of POMC neuronal activation is accompanied by an exaggerated anorexigenic response to exogenous melanocortins, suggesting a compensatory upregulation of downstream melanocortin response pathways in *Magel2*-null mice. The role of MAGEL2 in melanocortin-associated neuronal pathways may provide important insights into dysfunctional ingestive behavior and obesity in Prader-Willi syndrome.

Insensitivity to peripheral leptin has been demonstrated in diet-induced and genetic models of obesity [44]–[47]. In principle, a failure to respond to acutely or chronically elevated leptin could be caused by reduced transport across the blood brain barrier, or by an intrinsic defect in leptin-responsive neurons. In the latter case, leptin insensitivity could be caused by failure of leptin either to inhibit the orexigenic drive (at NPY neurons), or to activate the anorexigenic drive (through POMC neurons), or both mechanisms, as in congenital leptin insensitivity in mice carrying an inactive form of the leptin receptor (*LepR^db^* mice). Although the anorexic response to peripherally administered leptin is absent in the *Magel2-*null mice, the electrophysiology results demonstrate that many arcuate hypothalamic neurons that express the leptin receptor remain leptin-sensitive. Specifically, *Magel2-*null ARC slices have a similar proportion of neurons displaying inhibitory responses to leptin as do slices from control animals, and these responses are of similar amplitude. Moreover, the remaining POMC neurons retain sensitivity to NPY, so the loss of the leptin-mediated excitatory response is not indicative of a global cellular defect within the ARC. This retention of leptin-mediated inhibitory responses is consistent with the modest level of obesity in *Magel2-*null mice compared with leptin-deficient *Lep^ob^* or leptin receptor null *Lepr^db^* mice. We did not test the response of VMN neurons to NPY in *Magel2*-null animals here.

Several mouse strains have been constructed in which leptin signaling pathways are selectively impaired in POMC neurons. Mice engineered without leptin receptor expression only in POMC neurons are mildly obese, with a significant increase in fat mass [48], [49], similar in magnitude to that previously reported in *Magel2-*null mice [18]. A similar degree of obesity and adiposity is seen in mice with inactivation of STAT3 in POMC neurons [23]. Unlike the *Magel2*-null mice, the POMC-STAT3 mutants remain sensitive to peripheral leptin, but they display defects in compensatory refeeding following food deprivation leading to reduced weight regain, similar to what we have observed in *Magel2-*null mice. Though the largely glutamatergic neurons of the VMN [50] remain leptin-responsive in the mutants, the loss of leptin signaling in other hypothalamic neurons in *Magel2*-null mice could underlie their more severe insensitivity to peripherally administered leptin. Rapid effects of leptin action on ARC leptin receptors have been linked to increased phosphatidyl inositol-3-kinase (PI3K) signaling [51], [52]. Accordingly, pharmacological blockade of PI3K signaling inhibits leptin-induced activation of POMC neurons [53]. Targeted deletion of PI3K signaling in POMC neurons also eliminates leptin-induced activation of POMC neurons, and significantly blunts the reduction in food intake provoked by intracerebroventricular leptin administration [53]. Interestingly, these mice do not appear to have any defects in weight gain or body composition, though a different strategy aimed at the downregulation of PI3K in POMC neurons does lead to a modest obesity phenotype and increased sensitivity to diet-induced obesity [54]. Investigations of a possible role of *Magel2* in PI3K signaling are thus warranted.

The complete absence of a physiological response to leptin in *Magel2-*null mice could have several causes. First, the *Magel2*-null mice catch up in weight compared to control and start accumulating excessive fat mass after weaning onto a standard chow diet, albeit at a modest rate. The resulting hyperleptinemia could contribute to systemic leptin resistance through a mechanism unrelated to or secondary to defective POMC neuron activation, but in any event caused ultimately by loss of *Magel2* function. Secondly, only half the normal number of ARC neurons expressed pSTAT3 in the ARC of *Magel2*-null mice after peripheral leptin treatment, and fewer *Magel2*-null neurons were activated by leptin as measured by c-fos expression. Third, *Magel2*-null mice had fewer POMC ARC neurons, and the remaining POMC neurons were not activated by leptin. The loss of excitatory leptin signaling at POMC neurons and their increased adiposity are consistent with a loss of key actions of leptin at ARC POMC and potentially other neurons in the *Magel2*-null mice [48], [49]. While our findings demonstrate a crucial role for POMC in the *Magel2*-null phenotype, the ARC contains a heterogeneous population of leptin-activated neurons it remains possible that the leptin-mediated activation of these neurons is also affected by loss of *Magel2* [20].

Intracellular responses to leptin receptor activation are mediated by a complex signaling cascade in POMC neurons [55], and this process is similar but not identical in other leptin-responsive neurons. For example, in leptin-activated neurons in the ventromedial nucleus (VMN), some neurons depolarize in response to leptin, some cells hyperpolarize, and the majority of cells do not respond to leptin administration [39], [56], [57]. The identical rates of leptin responsiveness in VMN of *Magel2*-null and control mice indicates that Magel2 is required for depolarizing responses in some neuronal subtypes but not in others. Likewise, the relative increase in the number of neurons expressing pSTAT3 in the VMN of leptin-injected compared to saline injected mice did not differ between genotypes.

Fasting in rodents induces a state of negative energy balance that is reflected by dramatic decreases in circulating leptin levels [1], [58], [59] and compensatory hyperphagia on re-feeding. Deficiencies in fasting-induced hyperphagia and compensatory weight gain are found in models of POMC neuron degeneration or in POMC-specific STAT3 mutant mice [22], [23]. Thus, appropriate regulation of POMC neurons in the ARC is critical to normal responses to food deprivation, which are clearly impaired in *Magel2*-null mice. Other hypothalamic pathways could also contribute to dysfunctional feeding behavior in *Magel2*-null mice. For example, orexin neurons normally activate NPY and inhibit POMC neurons to stimulate increases in food intake [60], and ablation of orexin neurons in the lateral hypothalamus causes a loss of fasting-induced arousal and defense of body weight during fasting [61]. In fact, *Magel2-*null mice have fewer orexin neurons and a significant reduction in hypothalamic levels of orexin-A [43], [62], which could contribute to the impaired compensatory hyperphagic responses in the *Magel2* null mice. In addition, there may be developmental defects in axonal outgrowth and synaptic contacts with other neurons in the remaining POMC, orexin, and other neuronal subtypes that require Magel2 developmentally, further impairing their leptin-mediated excitability.

Notably, the anorexic response to melanocortins is intact and hyperactivated in *Magel2*-null mice, suggesting that melanocortin receptors in the paraventricular nucleus and elsewhere in the central nervous system are not impaired by loss of Magel2. Further examination of melanocortin responsiveness in *Magel2-*null mice could provide compelling evidence for potential therapeutic intervention in PWS. The exact biochemical roles of Magel2 and how it participates in neuronal differentiation and/or survival as well as cellular activation in response to leptin remain to be determined. In summary, our results demonstrate that Magel2 is critical for leptin responses in POMC neurons in the ARC and for energy homeostasis in mice. Further experiments are required to determine whether this defect is degenerative in nature or whether mice lacking *Magel2* are congenitally leptin insensitive. It will also be important to address whether loss of MAGEL2 in people with PWS likewise contributes to disrupted ingestive behavior and energy homeostasis in this disorder.

## Materials and Methods

### Mouse Strains

All animal procedures were approved by the University of Alberta Animal Care and Use Committee in accordance with the guidelines of the Canadian Council on Animal Care. Mice were weaned between 3–4 weeks of age and then group housed 3–5 per cage with food (PicoLab Rodent Diet 5001) and water *ad lib.*, and housed under a 12∶12 light∶dark cycle.

*Magel2*-null mice were generated [43] and housed [19] as described, and are available from the Jackson Laboratories (C57BL/6-Magel2tm1Stw/J, stock 009062). To identify specific neuronal populations, *Magel2* ^−m/+p^ carrier males were crossed with homozygous LepRb^EGFP^ reporter mice, which express enhanced green fluorescent protein (EGFP) in LepRb+ cells [63], or homozygous POMC^EGFP^ reporter mice, expressing EGFP in POMC+ cells (The Jackson Laboratory stock #009593, Bar Harbor, Maine) [33]. This cross produces *Magel2*×LepRb^EGFP^ or *Magel2*×POMC^EGFP^ mice, lacking *Magel2* but expressing LepRb^EGFP^ or POMC^EGFP^, and control littermates expressing wildtype *Magel2* and the reporter gene.

### Food Withdrawal and Refeeding

Male (12–16 weeks) mice were singly housed for at least one week, then weighed and fasted for 48 h beginning at 1600 h. Body weight was recorded 24 h and 48 h later, and food intake and body weight change were measured during 3 days of refeeding.

### Leptin and Melanocortin Sensitivity

Mice were singly housed for at least one week before the start of the experiment. One week before drug injections, mice were injected daily for 3 days with phosphate buffered saline, pH 7.3 (PBS). Body weight and food intake were measured during this time. On experimental day 1, food was removed at 1500 h and mice injected intraperitoneally (ip) with either 2.5 mg/kg mouse recombinant leptin (Dr. A.F. Parlow, National Hormone and Peptide Program, NHPP-NIDDK, Torrance, California), 2.5 mg/kg synthetic melanocortin agonist melanotan-II (MT-II Phoenix Pharmaceuticals, Burlingame, California) [64], or PBS at 1600 h. Food intake was measured 2–24 h later. After a 3 day recovery, the experiment was repeated using a cross-over design.

### Leptin Stimulation and Immunohistochemistry

Adult (12–16 weeks) mice were handled daily for 2 weeks, including one week of PBS injections, to minimize injection-related c-fos responses in the brain. Mice were then injected ip with either 2.5 mg/kg leptin or PBS 45 min before terminal pentobarbital anesthesia, paraformaldehyde perfusion, and preparation of coronal 30 µm hypothalamic sections for immunohistochemistry (IHC). For pSTAT3 IHC, sections were pretreated with 1% NaOH and 1% H_2_0_2_ in H_2_0 for 20 min, 0.3% glycine in PBS for 10 min, and 0.03% sodium dodecyl sulfate in PBS for 10 min, blocked for 1 h with 3% normal goat serum in PBS/0.3% Triton X-100, then incubated overnight with anti-pSTAT3 antibody (1∶1000, 9131, Cell Signaling, Danvers, Massachusetts). For c-fos and POMC/EGFP IHC, sections were washed in PBS, blocked for 30 min in 3% normal serum in PBS/0.3% Triton X-100, then incubated for 48 h with primary antibody (c-fos (Ab-5), 1∶2000, PC-38, Millipore, Billerica, Massachusetts; POMC, anti-GFP, 1∶4000, ab13970, AbCam, Cambridge, Massachusetts). After primary incubation, sections were washed with PBS/0.3% Triton X-100 and incubated for 2 h with goat anti-rabbit secondary antibody (Alexa Fluor 594) or goat anti-chicken secondary antibodies (Alexa Fluor 488) (1∶500, Invitrogen, Carlsbad, California), slide-mounted then imaged using a Zeiss LSM510 confocal microscope. For cell counting in the arcuate nucleus, sections were organized in a rostral to caudal manner through the hypothalamus according to the mouse brain atlas (www.mbl.org). Cells were counted using MetaMorph Imaging Suite (Molecular Devices, Sunnyvale, California) for pSTAT3, and Image J (National Institutes of Health, Bethesda, Maryland) for c-fos and GFP.

### Brain Slice Preparation and Electrophysiology

Brains from 6–12 week old male and non-estrous female reporter mice were prepared for patch clamp electrophysiology [39]. Slices were incubated for at least 1 h at room temperature in carbogenated artificial cerebrospinal fluid (aCSF) containing (in mM): 124 NaCl, 3 KCl, 1.3 MgSO_4_, 1.4 NaH_2_PO_4_, 2.5 glucose, 7.5 sucrose, 26 NaHCO_3_ and 2.5 CaCl_2_ (300–305 mOsm/L). For electrophysiology, slices were continuously perfused (2–4 ml/min) with warm (32–34°C), carbogenated aCSF. Cells expressing GFP were identified by epifluorescence illumination, then the light source was switched to infrared-differential interference contrast imaging to obtain whole-cell recordings. Visualized-patch whole-cell recordings were obtained using thin-walled glass patch pipettes with resistances of 5–7 MΩ when backfilled with an internal solution containing (in mM): 126 K-gluconate, 4 KCl, 10 HEPES, 5 MgATP, 0.3 NaGTP, 1 EGTA, 0.3 CaCl_2_ and 0.02% neurobiotin (pH adjusted to 7.25 with KOH, 280 mOsm/L). Stock solutions were prepared in PBS, pH 7.8 (leptin) or HPLC grade water (human NPY, Peptidec Technologies Ltd., Pierrefonds, Quebec, Canada), then diluted into aCSF immediately before use, and gravity-perfused into the recording chamber for at least 3 min. Slices were washed with aCSF for at least 10 min between drugs. A stable and reversible change in membrane potential of at least 2 mV from baseline appearing within minutes after drug application was considered a valid pharmacological response.

### Statistical Analysis

Statistical analyses were performed using a Student's unpaired *t*-test or a Fisher's Exact Test (GraphPad, La Jolla, California), with differences with *P*<0.05 after correction for multiple *t*-testing considered significant.
